# Take-off engine particle emission indices for in-service aircraft at Los Angeles International Airport

**DOI:** 10.1038/sdata.2017.198

**Published:** 2017-12-19

**Authors:** Richard H. Moore, Michael A. Shook, Luke D. Ziemba, Joshua P. DiGangi, Edward L. Winstead, Bastian Rauch, Tina Jurkat, Kenneth L. Thornhill, Ewan C. Crosbie, Claire Robinson, Taylor J. Shingler, Bruce E. Anderson

**Affiliations:** 1 NASA Langley Research Center, Hampton, VA 23681, USA; 2 Science Systems and Applications, Incorporated, Hampton, VA 23666, USA; 3 Deutsches Zentrum für Luft- und Raumfahrt (DLR), Stuttgart 70569, Germany; 4 Deutsches Zentrum für Luft- und Raumfahrt (DLR), Oberpfaffenhofen, 82234 Weßling, Germany; 5 NASA Postdoctoral Program, Columbia, MD 21046, USA

**Keywords:** Energy and society, Environmental monitoring, Atmospheric chemistry

## Abstract

We present ground-based, advected aircraft engine emissions from flights taking off at Los Angeles International Airport. 275 discrete engine take-off plumes were observed on 18 and 25 May 2014 at a distance of 400 m downwind of the runway. CO_2_ measurements are used to convert the aerosol data into plume-average emissions indices that are suitable for modelling aircraft emissions. Total and non-volatile particle number EIs are of order 10^16^–10^17^ kg^−1^ and 10^14^–10^16^ kg^−1^, respectively. Black-carbon-equivalent particle mass EIs vary between 175–941 mg kg^−1^ (except for the GE GEnx engines at 46 mg kg^−1^). Aircraft tail numbers recorded for each take-off event are used to incorporate aircraft- and engine-specific parameters into the data set. Data acquisition and processing follow standard methods for quality assurance. A unique aspect of the data set is the mapping of aerosol concentration time series to integrated plume EIs, aircraft and engine specifications, and manufacturer-reported engine emissions certifications. The integrated data enable future studies seeking to understand and model aircraft emissions and their impact on air quality.

## Background & Summary

Aircraft engine particle emissions are important contributors to local air quality near airports^1–5^, and these downstream environmental impacts are likely to increase in concert with the projected growth of the aviation sector over coming decades. Emissions relevant to local impacts occur during multiple stages of aircraft movement including idle, taxi, take-off, and the portion of climb out and landing below 3,000 feet altitude above field elevation. Collectively these stages are referred to as the landing and take-off (LTO) cycle. Emissions standards for each phase of the LTO cycle are set by the International Civil Aviation Organization (ICAO) pursuant to Volume II of Annex 16 of the Convention of International Civil Aviation^6^, which recommends standard engine emissions testing methods for gas-phase and aerosol species. Aerosol emissions are quantified by engine manufacturers prior to certification and operation in terms of a smoke number metric that describes how soot particles collected by a filter change the reflectance of the filter over a defined sampling time, and which is known to be dependent on sampling conditions and soot properties.

Recognizing the significant limitations of the smoke number metric, current and future efforts are underway to measure engine LTO aerosol emissions in a more rigorous fashion by measuring particle number and/or mass emission indices. While these data will inform transportation modelling of the next generation of aircraft engines, there are currently no plans to recertify older engines that are in service now (and are likely to remain in service in the coming decades). In addition, the LTO emissions certification process is idealized as engine conditions are measured under discrete, steady thrust settings that may differ from the thrust actually applied by pilots. For example, thrusts applied during taxi and idle may be lower than the 7% of maximum thrust that is commonly assumed. Similarly, pilots frequently take off under reduced thrust of up to 25% below maximum, depending on runway conditions and aircraft weight and specifications^7,8^. Congestion on taxiways may lead to sudden engine accelerations and decelerations as aircraft taxi to and from runways and thrust reversers may be applied during landings, all of which are non-standard conditions^5^. In addition, aerosol particles undergo modifications by condensation and coagulation in the airport environment. These modifications lead to effective emissions that impact airport air quality not considered in the ICAO certification process. Finally, the amount of time spent in each phase of the LTO cycle (referred to as time-in-mode, or TIM) drives the overall amount of engine emissions and is not well constrained.

**Figure 1:**
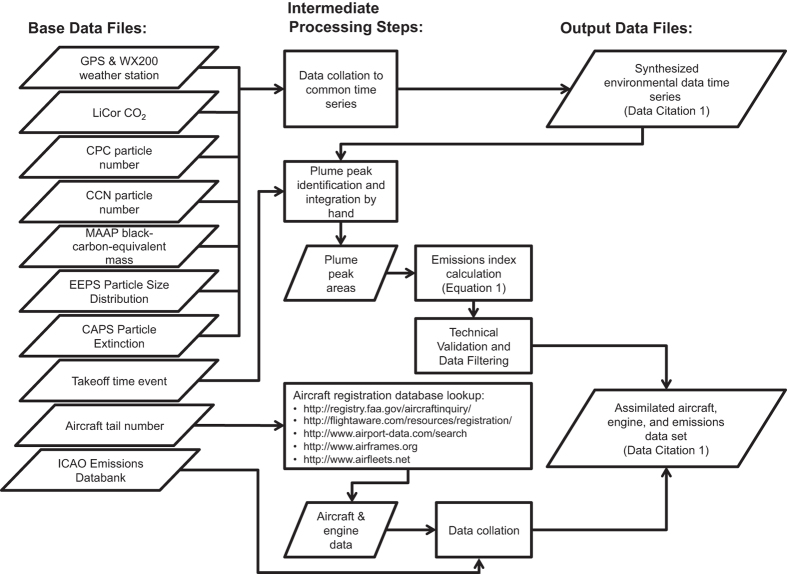
Workflow from base data file generation to processing, analysis, and output data set production.

Given these myriad sources of variability, there is a need to understand the emissions from currently in-service engines under real-world conditions. Here, we investigate particles emitted by aircraft during take-off operations at Los Angeles International Airport and synthesize these emissions measurements with information on the aircraft and engine specifications as well as the ICAO certification emissions values for each engine model. The work flow of this study is shown in Fig. 1, leading from the base data files generated by the instruments, field notes, and existing emissions databank, through the intermediate analysis and processing steps, to arrive at synthesized output data files that form the two data records described by this data descriptor. The environmental, aircraft, engine, and emissions data parameters are listed in Table 1.

This comprehensive dataset informs future studies seeking to model the impacts of take-off engine emissions at and in the vicinity of airports, to evaluate the performance of current approximation methods for estimating emissions from smoke number measurements, to explore plume-averaged and transient emissions profiles during many aircraft take-off events, and to understand the background aerosol concentrations and properties downwind of a major airport in between the take-off events, among other uses. A powerful and unique feature of this data set is that ambient concentrations of both aerosols and carbon dioxide are used to compute EIs and link these EIs to specific aircraft and engines as well as engine specifications and emissions standards.

## Methods

### Study site

**Figure 2:**
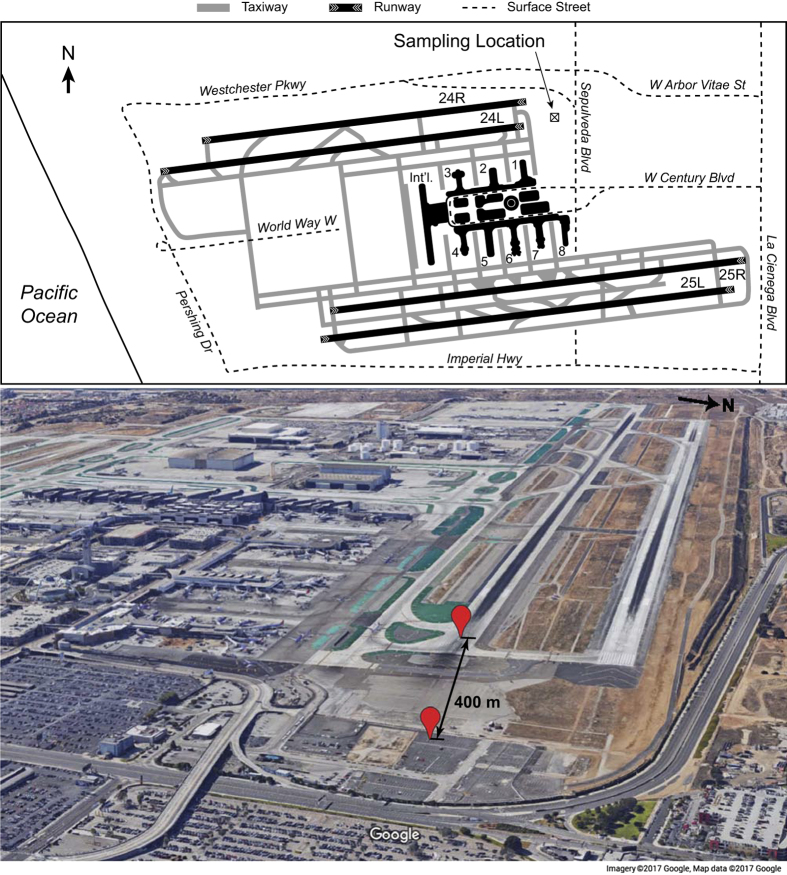
Los Angeles International Airport schematic and aerial view. The sampling location is indicated by the [X] icon in the employee parking lot just west of Sepulveda Boulevard and downwind of take-off runway 24L. Red markers denote the sampling location and the beginning of Runway 24L. The distance between the two points is about 400 m.

Data were collected at 400-m distance downwind of the northern take-off runway (24L) of Los Angeles International Airport (33.9509°N, 118.398°W) on two days: 18 May and 25 May 2014. A schematic of the airport and aerial view of the sampling location near the runway are shown in Fig. 2. Runway 24L has a declared length of 3,135 m, 263° true bearing, and is at an elevation of 38 m. A predominantly onshore sea breeze of 0–10 m s^−1^ was oriented down the runway (±20°) during both measurement days, which advected the aircraft take-off plumes to the sampling inlet of the NASA Langley Aerosol Research Group (LARGE) Mobile Laboratory. The height of the inlet was approximately 3 m above the ground. Ambient temperature and relative humidity varied between 20–25 °C and 45–65%, respectively. Table 1 shows the complete list of environmental data.

Ambient air was drawn through a 1.3 cm outer diameter stainless steel tube and distributed to instruments inside the mobile laboratory with varying transport tubing lengths of 6–10 m depending on where each instrument was mounted. Given the uncertainty in these varying transmission lengths, the data are not corrected for size-dependent diffusional losses to the tubing walls. As a constraint on these impacts, assuming a nominal flow rate of 50 l min^−1^, calculations suggest that 13–22% of 20-nm-diameter particles are lost across the full range of transport tubing lengths^9^.

### Fuel properties

Laboratory analyses of fuel composition were obtained from LAXFUEL for the tanks on issue on May 18th and 25th, which describe the fuel sulphur content, aromatic content, naphthalenes content, and the net heat of combustion. Mean fuel properties (±1 standard deviation) are reported for each day in Table 2, assuming equal weighting across the two fuel batches analysed for the 18th and across the four fuel batches analysed for the 25th. On the 18th only a single tank was on issue, while on the 25th, a tank fuel containing moderate sulphur and low aromatics (710 ppmm S and 12 % by volume, respectively) was initially on issue and was followed by another tank containing a blend of domestic and foreign fuels whose sulphur and aromatic contents varied considerably (620–1,780 ppmm S and 17.6–23% by volume, respectively). Since it is not possible to determine when a particular aircraft would have fuelled prior to departure, it is not possible to draw composition-specific conclusions on emissions parameters. For completeness, we report here the individual batch analyses and the daily-averaged fuel properties, which are indicative of moderate sulphur content and typical aromatic content as compared to previously reported jet fuel properties^10,11^.

### Instrumentation and measurement methods

Environmental and emissions measurement parameters are summarized in Table 1, and a brief description of each instrumental measurement method is given below.

#### Aircraft tail numbers, take off times, and plume start times

The time of each aircraft take off event and the aircraft tail number were recorded by an observer in the field. These data were then matched to a plume peak in the measurement time series. Of these pieces of information, the time recorded in the field when the observer noticed the aircraft begin to take off (TakeoffStartTime_UTC) can be uncertain because the observer was distracted, troubleshooting instruments, or missed the start of take off and instead wrote down the time when the measurement peak appeared. Consequently, while these times are used to roughly match the aircraft tail numbers to observed concentration peaks, we also report PlumeStartTime_UTC as the start of the plume as determined during data post-processing. In a few instances, TakeoffStartTime_UTC is after PlumeStartTime_UTC, and the latter is the more reliable data parameter.

#### Meteorological parameters

Temperature, pressure, wind speed, and wind direction were measured continuously with a WeatherHawk 232 weather station mounted on top of the mobile laboratory near the sampling inlet. Air temperature is measured to within ±0.5 °C accuracy with a thermistor, while the piezoresistive barometric pressure transducer is accurate to within ±1.5 kPa. Wind speed is measured with a cup anemometer with a starting threshold of 0.78 m s^−1^, and wind direction is determined from a vane sensor with 1% linearity and ~1 m s^−1^ sensitivity.

#### Carbon dioxide (CO_2_)

CO_2_ mixing ratio was measured at 1 Hz with a Licor LI-7000 CO_2_/H_2_O Gas Analyser. The instrument measures the differential absorption of light by carbon dioxide and water vapour at 4.255 and 2.595 μm wavelengths, respectively. Only the CO_2_ data were calibrated and are included in this data set.

#### Black-carbon-equivalent particle mass (BC)

BC mass concentration was measured with a Thermo Scientific Multi-Angle Absorption Photometer (MAAP)^12^. The MAAP continuously measures the amount of light transmitted through a particle-loaded glass fiber filter material as well as light backscattered off of the filter, both at 670 nm wavelength. These measurements are then used to determine a black carbon equivalent aerosol mass concentration by assuming a mass absorption coefficient of 6.6 m^2^ g^−1^. The instrumental uncertainty is estimated to be ±12%^13^.

#### Particle number

Particle number concentration was measured at 1 Hz with a TSI Condensation Particle Counter (CPC; Model 3775). For particle concentrations less than 5×10^4^ cm^−3^, individual particles are detected as pulses of laser light (‘single-counting mode’), while for higher particle concentrations (5×10^4^ to 1×10^7^ cm^−3^) the total amount of light scattered by the particle population is used to determine concentration (‘photometric mode’). Particle concentration accuracy is reported as±10% in single-counting mode and±20% in photometric mode. The minimum detectable particle size of the CPC is 4 nm diameter and it has a response time of 4 s.

#### Non-volatile particle number

Non-volatile particle number concentration was measured at 1 Hz with a TSI CPC (Model 3022A) located downstream of a thermal denuder. The thermal denuder is a stainless steel tube heated at 350 °C in order to evaporate volatile material on the aerosol (e.g., sulphur and nitrate species and organics). The 3022A CPC is similar to the 3775 Model, except that the minimum detectable particle size is 7 nm diameter and the response time is <13 s. This difference in lower detection size does not preclude direct comparison of total and non-volatile particle number because non-volatile particles, such as black carbon, are known to be greater than 10–20 nm in diameter.

#### Particle number and volume size distribution

The particle number size distribution between 6 and 575 nm diameter was measured at 1 Hz with a TSI Engine Exhaust Particle Sizer (EEPS; Model 3090). Particles are drawn into the EEPS at 10 l min^−1^ flow rate and are given a positive charge via corona charging before entering a measurement region of two concentric cylinders on which an electric field is applied. The positively-charged particles move toward the outer electrode at a rate proportional to their size-dependent electrical mobility until they ultimately impact on one of several sensitive electrometers. An inversion algorithm is applied to the time-dependent electrometer currents to retrieve the aerosol size and number concentration at 1 Hz.

Past comparisons between an EEPS (or similar instruments such as the Cambustion DMS500 and TSI FMPS) and a Scanning Mobility Particle Sizer (SMPS) have shown that the EEPS slightly undersizes soot particles relative to the SMPS^14–18^. This undersizing is particularly problematic for large, diesel soot agglomerates, and is less of an issue for aircraft soot that tend to be sub-100-nm in diameter and closer to compact spheres than fractal agglomerates. In order to better understand the performance of the EEPS relative to the state-of-the-art SMPS, we examined data from both instruments during the NASA Alternative Fuel Effects on Contrails and Cruise Emissions (ACCESS) project, which sampled the exhaust of the NASA DC-8 CFM56 engines. This comparison showed that the EEPS undersizes particles by about 10% relative to the SMPS for intermediate and high engine thrust settings, which is similar to the size discrepancy reported by Hagen *et al.*^14^ for aircraft soot sampled during the 2004 NASA APEX project. Consequently, we have corrected the size bins by a scaling factor of 1.1 for all EEPS data, but also report the uncorrected EEPS size bin diameters in the data record files. The EEPS-SMPS comparison using ACCESS data shows good agreement between the number concentrations reported by both instruments, which are well within the previously reported instrumental uncertainty of ±20%^16^.

#### Cloud condensation nuclei (CCN) number

CCN concentration at (2.6±0.2)% water vapour supersaturation was measured at 1 Hz with a Droplet Measurement Technologies CCN Counter^19,20^. The instrument was operated at an elevated flow rate of 1 l min^−1^ and elevated temperature gradient of 16 °C to effect this high supersaturation, which was calibrated using size-classified, dry ammonium sulfate aerosols and Scanning Mobility CCN Analysis^21^. The uncertainty in supersaturation of 0.2% is propagated from the scatter in the calibration critical activation diameters using Köhler theory with corrections for incomplete solute dissociation following the ion-interaction approach of Pitzer and Mayorga with parameters obtained from Clegg and Brimblecombe^22–24^. The uncertainty in the CCN concentration is estimated to be 7–16%^25^.

#### Particle extinction coefficient

Aerosol light extinction is measured at 530 nm wavelength with a cavity attenuated phase shift extinction (CAPS PMex) monitor^26^. The instrument sensitivity is 2.5 Mm^−1^ with a response time of less than two seconds. The CAPS PMex instrument is also sensitive to the presence of absorbing gases (e.g., NO_2_), which were not measured during this project. Yu *et al.*^27^ found this correction to relatively minor for aircraft engine idle conditions, and the emissions index of NO_2_ at higher engine thrusts is even lower than at idle^28^.

### Emission index calculation

Here, we report engine emissions parameters in terms of a plume-average emissions index that is normalized to the rate of engine fuel burn. This normalization process takes into account differences in plume dilution that can be affected by turbulence, varying wind speed and direction, as well as differences in sampling instrument response time constants. For example, the MAAP response time is much slower than that of the particle concentration and size distribution measurements owing to the internal averaging and smoothing algorithms applied by the instrument firmware.

The emission index of particle species X is determined following Moore *et al.*^29^ as

$${\mathrm{EI}}_{X}=\frac{X}{{\text{CO}}_{2}}\;\frac{V_{m}}{M_{{\mathrm{CO}}_{2}}}\left({\mathrm{EI}}_{{\mathrm{CO}}_{2}}\right)$$

$$\mathrm{where},\;{\mathrm{EI}}_{{\mathrm{CO}}_{2}}=\frac{RT}{PV_{m}}\;\frac{M_{{\mathrm{CO}}_{2}}}{\left(M_{C}+\alpha M_{H}\right)}∼3160\;{\mathrm{gCO}}_{2\;}\;{\text{kg}}^{-1},$$

ΔX and ΔCO_2_ are the background-subtracted peak areas of the measured concentrations of species X and CO_2_ at standard temperature and pressure, respectively; EI_CO2_ is the emissions index of CO_2_, assuming that the carbon content in the fuel is constant and is completely converted to CO_2_; *R* is the ideal gas constant; *T* is the temperature at STP (273.15 K); *P* is the pressure at STP (1 atm); *V*_*m*_ is the molar volume of ideal gas at STP (22.4 l mol^−1^); α is the fuel hydrogen-to-carbon molar ratio (assumed to be 1.92); and M_CO2_, M_C_, M_H_ are the molar masses of CO_2_, carbon, and hydrogen, respectively.

**Figure 3:**
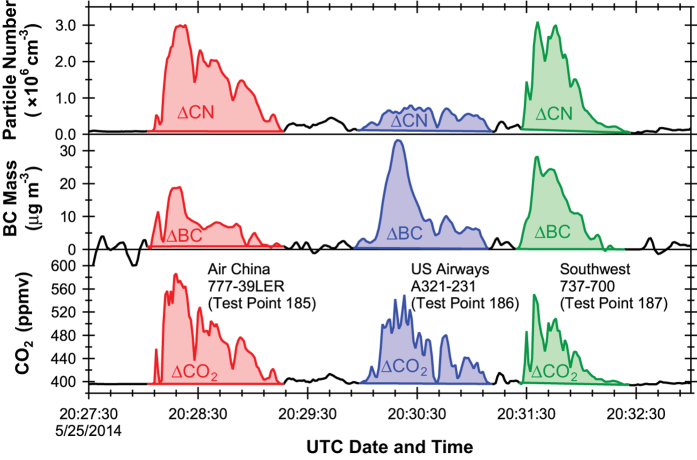
Example time series showing the integrated background-subtracted plume peak areas. Measurements shown are carbon dioxide mixing ratio (ΔCO_2_), MAAP black carbon mass concentration (ΔBC), and CPC total particle number concentration (ΔCN) during three take off events (Test Points 185–187).

Figure 3 shows example time series of CO_2_, black carbon mass concentration (BC), and particle number concentration (CN), where the shaded regions represent the background-subtracted peak areas (ΔCO_2_, ΔBC, and ΔCN). Bounding points were visually set by the authors on either side of the peak and a linear fit between those points establishes the background baseline. The background-subtracted peak area is then the difference between the integrated area under the concentration time series and the area under the background baseline between those two points. This was determined using the areaXY function in Igor Pro (Wavemetrics, https://www.wavemetrics.com/).

### Ancillary data sets

#### Aircraft registration

The United States Federal Aviation Administration (FAA) and similar national regulatory agencies maintain civil aircraft registration records that describe the general specifications of the airframe and engines as well as attesting to the ownership of the aircraft and its airworthiness. Each aircraft has a unique tail number identifier that maps to the various national registration databases, with the first letter denoting the nationality of the aircraft. For example, the United States nationality designator is ‘N’, and U.S. flag aircraft have tail numbers that begin with ‘N’ followed by a unique alphanumeric identifier. The aircraft tail numbers were photographed with a telephoto lens prior to take-off as the plane taxied to the runway. The tail number was then used with aircraft registration databases as in Fig. 1 to obtain the detailed specifications of the aircraft and engine. These data include the airline (also visible from the aircraft markings), the aircraft manufacturer and year of manufacture, and the aircraft model and series. In addition, the number of engines, their manufacturer, and their model and series are contained within the registration database. A list of compiled aircraft and engine parameters is given in Table 1, and the emissions data are summarized by engine type in Table 3.

#### ICAO aircraft engine emissions databank (EDB)—Version 23b

The International Civil Aviation Organization maintains a database of the engine exhaust emissions parameters of production aircraft engines. Emissions are characterized by the engine manufacturers following ICAO Annex 16 Vol II and reported on a voluntary basis^30^. For each engine model and series, the EDB lists specifications including the engine type (for example, turbofan, mixed turbofan, and turboprop), the engine bypass and pressure ratios, and the engine maximum rated thrust. Emissions parameters reported in the EDB include carbon monoxide, nitrogen oxides, hydrocarbons, and smoke number for four different engine power conditions that correspond to different point in the LTO cycle (idle, approach, climb out, and take off conditions). Detailed information about the test-specific fuel, its properties, and fuel flow rates at each power setting are also provided. Since this data descriptor focuses on aerosol emissions, we only include the EDB smoke number and associated test and fuel information in the synthesized data set (Data Citation 1).

### Code availability

Data were analysed with commercially-available software including Igor Pro 6.37 and Microsoft Excel 2013. Summary statistics reported in the Technical Validation Section in this data descriptor are generated with custom code in ‘R’, which is available without restriction in the data records as an HTML file: LAX-Ground-ProcessingCode_R01.html.

## Data Records

Two identical data records are associated with this work: a set of data archived in the NASA Aeronautics Field Projects database (https://aero-fp.larc.nasa.gov/projects/lax) and a Dryad data set (Data Citation 1). While the NASA database is the primary repository for NASA Aeronautics emissions research, the Dryad database is indexed with a digital object identifier (DOI) ensuring a persistent identifier is assigned to the data set. Each data record contains two files corresponding to the Output Data Files in Fig. 1. Time series of synthesized aerosol and carbon dioxide concentration data on a common time base are found in the LAX-Ground-Summary_TS_20140518_R01_thru20140525.xlsx Microsoft Excel workbook, where TS denotes time series data. Meanwhile, the calculated emissions indices across both days are assimilated with aircraft- and engine-specific parameters for each of the 275 take-off plume test points in the Microsoft Excel workbook entitled LAX-Ground-Summary_TP_20140518_R01_thru20140525.xlsx, where TP denotes test point data. Also included in both data records is an HTML file containing code used to process the data and figures in ‘R’ for this data descriptor named LAX-Ground-ProcessingCode_R01.html.

Archived measurement parameters and associated aircraft and engine characteristics are detailed in Table 1. These data map to the README tabs in the.xlsx workbooks.

The test points cover 29 different aircraft engine master model combinations that correspond to a wide variety of aircraft as shown in Table 3. The majority of aircraft plumes sampled come from CFM56-3B and CFM56-7B class engines that are popular in the Southwest fleet of 737s, from the CFM56-5B class engines on Virgin America Airbus A319/320/321 aircraft, and from GE CF34-8 class engines on Delta Connection CRJ and ERJ aircraft. This distribution is due, in part, to the close proximity of Runway 24L to Terminal 1, which serves Southwest Airlines.

## Technical Validation

**Figure 4:**
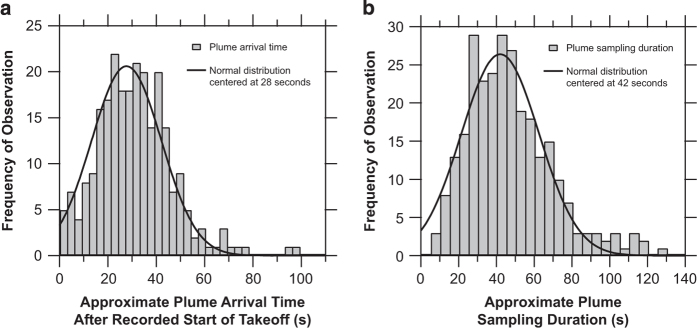
Take-off plume elapsed time and duration histograms. Reported are the frequency of occurrence of the approximate elapsed time between the observed start of aircraft take-off and increase in measurement parameters (**a**), and the duration of the sampled plume (**b**).

**Figure 5:**
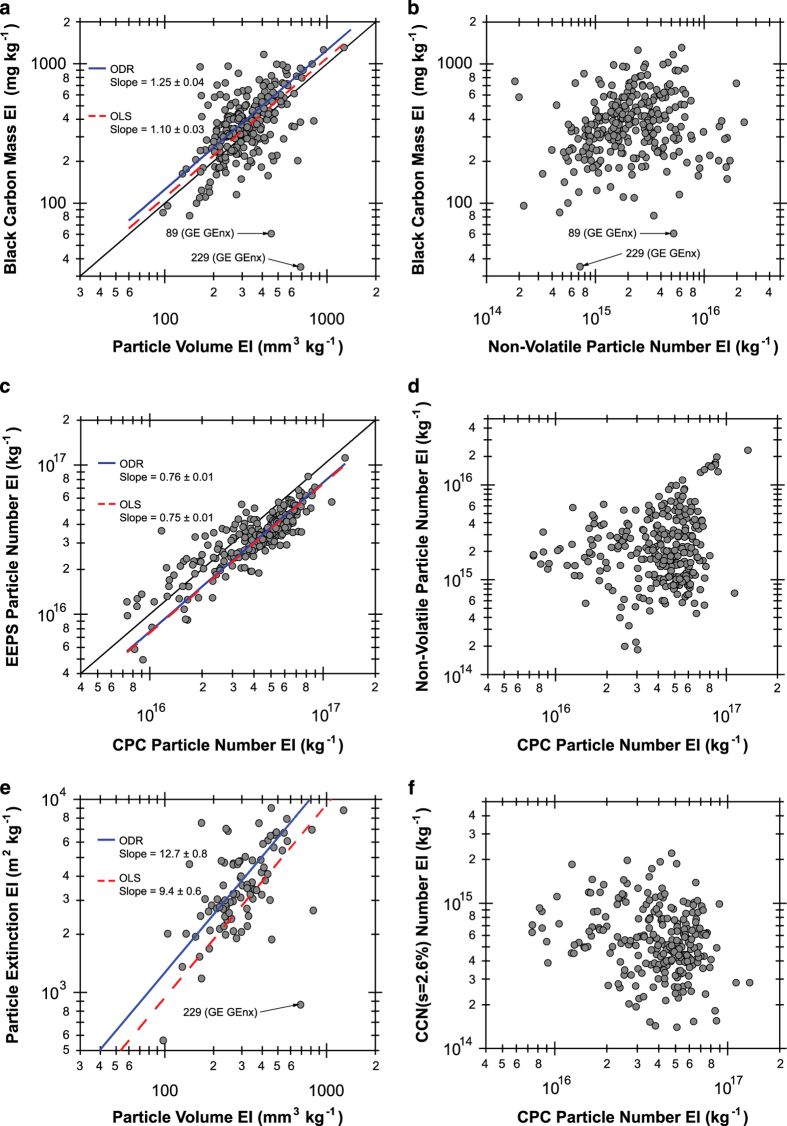
Scatter plots showing the relationship between measured particle emissions indices (EIs). Solid lines are orthogonal distance regression (ODR) linear fits to the data, while dashed lines are ordinary least squares (OLS) linear fits. For both fits, the intercept was held at zero. Outlier test points 89 and 229 are noted, which correspond to the two plume intercepts of the GE GEnx engine. Note that kg^−1^ denotes ‘per kilogram of fuel’.

**Figure 6:**
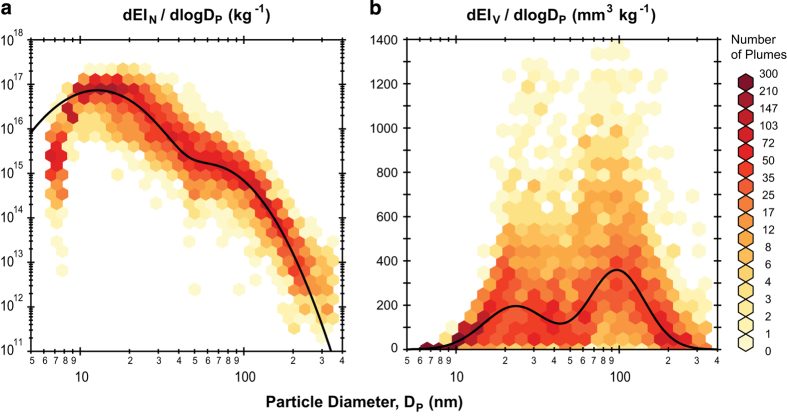
Size distributions of take-off particle number and volume emissions indices. Each of the 275 sampled plumes are binned as denoted by the colored hexagons for number EI (**a**) and volume EI (**b**). The solid lines are two-mode, log-normal fits to the geometric mean distribution of all plumes for which size distribution data are available (*N*=253) with fit coefficients given in Table 5.

Data were compiled into the data records described above, and data integrity is verified as follows:

1. Plume peaks in the time series data are matched to specific aircraft take-off events as recorded in the field during 18 and 25 May. The histogram in Fig. 4a shows the typical elapsed time between the observed start of take-off and when the plume is detected by the instrumentation. Typical values vary between 10–45 s and depend on the engine thrust, ambient wind speed, and wind direction. Once the plume is detected, the typical duration is 40±20 s (Fig. 4b), which is in good agreement with the ICAO reference time-in-mode of 42 s for aircraft in the take-off portion of the LTO cycle^8,31^.
2. Each of the 275 background-subtracted plume peak areas is integrated using the areaXY function in Igor Pro with bounding background points set by the authors. The peak fitting procedure was carried out independently by a co-author in order to verify the accuracy of the resulting data. In general, the independently calculated EIs agree well with the data values reported here and were within 31, 3.4, and 14%, on average, for the BC mass, particle number, and non-volatile particle number EIs, respectively. A discrepancy of greater than 100% was found for only 18, 5, and 14 test points, respectively, which drives these uncertainties. Significant outliers were examined individually to ensure data quality. Common issues that were identified and fixed include errors in peak identification/attribution when several aircraft took off over a short period of time, MAAP baseline fluctuations that influenced the background subtraction, and transcription errors.
3. Scatter plots showing the relationships between the calculated EIs for each aircraft take-off plume are shown in Fig. 5. Where linear relationships between the two data variables are expected, blue, solid lines denote orthogonal distance regression and red, dashed lines denote ordinary least squares regression linear fits. Particle mass and volume are both dominated by larger black carbon, soot mode aerosols. As shown in Fig. 5a, the ratio of the optically-derived soot mass to the integrated size distribution volume constrains the soot effective density near unity, which is consistent with past measurements of aviation emissions particle density^32–34^. Two notable outliers are Test Points 89 and 229, which correspond to the GE GEnx engines. Unlike the other engines whose emissions were sampled, the GEnx line features a lean burn combustion system that appears to substantially reduce the black carbon mass emissions at take-off conditions; however, overall particle number and volume EIs are similar to those for other engine types. Good linearity is observed between particle number EIs derived from the CPC and the integrated EEPS size distribution (Fig. 5c) as well as between the CAPS particle extinction coefficient EIs and EEPS particle volume EIs (Fig. 5e). The 25% offset between the EEPS and CPC number is likely due to differences in the lower detection limits of each instrument (5.6 and 4 nm, respectively). No clear relationships are apparent between the other computed EI parameters.
4. Summary statistics for each of the 29 different aircraft engine master model types are given in Table 4 as the geometric mean EI times-divide (⋇) one geometric standard deviation (g.s.d.). Singular values without a reported g.s.d. indicate that only one plume was sampled for that engine type. Particle number EIs are of order 10^16^–10^17^ kg^−1^, non-volatile particle number EIs are of order 10^14^–10^16^ kg^−1^, and CCN number EIs are of order 10^14^–10^15^ kg^−1^. Meanwhile, geometric mean black-carbon-equivalent particle mass varies from 175–941 mg kg^−1^ (except for the GE GEnx engines at 46 mg kg^−1^) and particle volume varies from 124–617 mm^3^ kg^−1^.
5. These EIs are broadly consistent with the findings of Lobo *et al.*^35^, who report the range of particle number EIs at 4×10^15^–2×10^17^ and mass EIs of 100–700 mg kg^−1^ for aircraft taking off from Oakland International Airport. Similarly, Klapmeyer and Marr^36^ found mean number EIs in the range of 1.4×10^16^–6.8×10^16^ for different regional aircraft taking off from Roanoke Regional Airport. Our number EIs are higher than the mean value reported by Herndon *et al.*^37^ at Boston Logan Airport of (8.8±7.6)×10^15^ kg^−1^, as well as the range of values of 1.8×10^15^–4.6×10^15^ kg^−1^ reported by Herndon *et al.*^38^ at Atlanta’s Hartsfield Jackson Airport. We attribute these differences to the use of a 3022A CPC in both of the previous studies, which has a larger minimum size detection limit than the 3775 CPC used in this study to measure total particle number.
6. Log-normal fits to the geometric mean particle number and volume size distributions across all aircraft plumes sampled are shown in Fig. 6a and 6b, respectively, overlaid on density maps of all observed size distributions. The data were fitted using a bi-modal, log-normal function of the form
  $$\frac{d{\mathrm{EI}}_{i}}{d\;\log D_{p}}=\frac{i_{1}}{\sqrt{2\pi }\log\sigma_{i1}}\;\exp\left[\frac{{\left(\log D_{p}-\log D_{i1}\right)}^{2}}{2\log^{2}\sigma_{i1}}\right]+\frac{i_{2}}{\sqrt{2\pi }\log\sigma_{i2}}\exp\left[\frac{{\left(\log D_{p}-\log D_{i2}\right)}^{2}}{2\log^{2}\sigma_{i2}}\right]$$
  where *i* is the total particle number (N) or volume (V) in the first (*i*_1_) or second (*i*_2_) size modes; EI is the emissions index; Dp is the dry particle diameter; D_i1_ and D_i2_ are the geometric mean diameters of the first and second size modes, respectively; σ_i1_ and σ_i2_ are the g.s.d. of the first and second size modes, respectively. Fit coefficients for the number and volume size distributions are given in Table 5.
7. The number distribution is dominated by a nucleation mode centred between 10 and 20 nm, while the volume distribution is dominated by the larger soot mode centred between 70–120 nm. Both of these features and their magnitudes are consistent with past measurements of advected aircraft plumes^35,37,38^.

## Usage Notes

Summary statistics reported in this data descriptor are generated with custom code in ‘R’, which is available in the data records as an HTML file: LAX-Ground-ProcessingCode_R01.html. All files are self-explanatory with metadata in the README tabs of the .xlsx data files.

Some possible uses of this dataset include:

- Incorporation of engine-specific geometric mean EIs and size distribution fit coefficients into airport emissions modelling to understand the effect of take-off emissions and their impact at and in the vicinity of airports.
- Comparison of ICAO smoke number data and measured EIs to evaluate current approximation methods employed by models for parameterizing aircraft take-off emissions (e.g., FOA3, FOX)^39,40^.
- Examination of the aerosol and CO_2_ concentration time series data to determine if aircraft EIs change during the approximately 0.7 min time-in-mode. For example, in a few cases a burst of non-volatile soot particles was initially emitted by the engines, but this was not sustained during the rest of the take-off plume duration. Is this common across engine types or due to operational factors?
- Analyses of the aerosol and CO_2_ concentration time series data outside of the immediate take-off plume events to determine baseline aerosol concentrations in the vicinity of and downwind of the northern LAX aircraft terminals.

A key feature of this data descriptor is that ambient concentrations of both aerosols and carbon dioxide are used to compute EIs and link these EIs to specific aircraft. This level of specificity is uncommon in the past literature, where only ambient aerosol concentrations are often reported. Given the usefulness to modellers of reporting results in terms of an EI (in other words, in terms of the mass of fuel burned), we suggest that future studies should follow this approach. In addition to the methodology employed in this data descriptor, it is recommended that future aircraft emissions sampling studies should also

- measure NO_x_ and CO (and preferably also gaseous hydrocarbons) in order to infer the actual aircraft thrust setting based on established methods, as it is known that aircraft frequently take-off at reduced thrust relative to their maximum rated thrust setting^8^. NO_x_ is particularly important because its EI increases monotonically with engine thrust^28^.
- target newer engines with lean-burn combustors (e.g., GE GEnx) to validate the findings of this work that these engines emit substantially less black carbon aerosol on a mass basis than other engines at take-off, but that the particle number and volume emissions are similar to other engines.
- improve statistical sampling of CRJ and ERJ series aircraft that together account for more than 30% of U.S. flights in 2011^8^, and which are underrepresented in studies to date.
- report aerosol concentration and EI summary statistics as either percentiles or as geometric means and geometric standard deviations (as in this work). The use of arithmetic means and standard deviations is widespread throughout the literature and incorrectly implies that the lower uncertainty bound is close to, or even in some cases below, zero.

## Additional information

**How to cite this article:** Moore, R. H. *et al.* Take-off engine particle emission indices for in-service aircraft at Los Angeles International Airport. *Sci. Data* 4:170198 doi: 10.1038/sdata.2017.198 (198).

**Publisher’s note:** Springer Nature remains neutral with regard to jurisdictional claims in published maps and institutional affiliations.

## Supplementary Material



## Figures and Tables

**Table 1:** Measurement parameters and associated aircraft characteristics.

**Environmental data:**	**Aircraft & engine information:**	**Emissions measurements:**
Date	Aircraft tail number	Carbon dioxide (CO_2_) mixing ratio
Take-off start time	Airline	Black-carbon-equivalent particle mass
Plume start time	Aircraft manufacturer	Particle (D_p_>4 nm) number
Plume sampling duration	Year of manufacture	Non-volatile particle (D_p_>7 nm) number
Latitude and longitude	Aircraft model/series	Particle number size distribution
Field elevation	Aircraft master model	Particle (5.6 nm<D_p_<560 nm) number
Runway length	Aircraft master series	Particle (5.6 nm<D_p_<560 nm) volume
Temperature	Number of engines	Cloud condensation nuclei (CCN) number at (2.6±0.2)% supersaturation
Dew point	Engine manufacturer	Particle extinction coefficient at 532 nm wavelength
Relative Humidity	Engine model/series	
Pressure	Engine type (TF, MTF, TP)	
Wind speed	Engine bypass ratio	
Wind direction	Engine pressure ratio	
	Engine maximum rated thrust	
	Engine certification smoke number	

**Table 2:** Fuel properties of tanks on issue during the sampling period.

**Fuel Analysis Batch**	**Batch Volume (Megaliters)**	**Sulfur (ppmm)**	**Aromatics (volume %)**	**Naphthalenes (volume %)**	**Heat of Combustion (MJ kg**^**−1**^)
18 May 2014 Tank 6014, Batch 1	14.58	1,530	17.7	0.81	43.249
18 May 2014 Tank 6014, Batch 2	14.98	1,280	18.5	1.31	43.252
**18 May 2014 Mean**	—	**1,400±180**	**18.1±0.6**	**1.06±0.35**	**43.251±0.002**
25 May 2014 Tank 402, Batch 1	—	710	12	0.63	43.238
25 May 2014 Tank 609, Batch 1	16.34	620	17.6	0.63	43.245
25 May 2014 Tank 609, Batch 2	2.86	1,600	23	2.8	43.033
25 May 2014 Tank 609, Batch 3	5.25	1,780	22.6	2.2	42.984
**25 May 2014 Mean**	—	**1180±600**	**18.8±5.2**	**1.57±1.11**	**43.13±0.14**

**Table 3:** Summary of sampled aircraft engines and airframes.

**Engine manufacturer, model, and series**	**Aircraft manufacturer**	**Years of aircraft manufacture**	**Aircraft model and series**	**No. of plumes sampled**
CFM CFM56-3B	Boeing	1987–1997	737–300, 737–500	20
CFM CFM56-3C	Boeing	1998	737–400	1
CFM CFM56-5A	Airbus	1990–1998	A319–100, A320–200	11
CFM CFM56-5B	Airbus	1999–2013	A319–100, A320–200, A321–200	40
CFM CFM56-5C	Airbus	2001	A340–300	2
CFM CFM56-7B	Boeing	1998–2014	737–700, 737–800, 737–900	86
GE CF34-3	Bombardier	2000–2003	CRJ-200	6
GE CF34-8	Bombardier, Embraer	2006–2011	CRJ-700, CRJ-900, ERJ-170	23
GE CF6-80C2	Boeing	1989–2012	747–400, 767–300	6
GE GE90-94B	Boeing	2002–2003	777–200	2
GE GE90-115B	Boeing	2003–2012	777–300, 777–300	6
GE GEnx-2B67	Boeing	2012–2014	747–800	2
EA GP7270	Airbus	2010–2013	A380–800	3
PW JT8D	McDonnell Douglas	1987	MD-80	2
PW PT6A	Beech	1996	1900D	7
PW PW118	Embraer	1994–1999	ERJ-120	10
PW 150A	Bombardier	2004–2013	DHC-8	3
PW PW2000	Boeing	1984	757–200	1
PW PW4000	Airbus, Boeing	2002	A330–200, 767–300	2
Rolls-Royce AE3007	Embraer	1999–2007	ERJ-145	2
Rolls-Royce RB211	Boeing	1992–1993	747–400, 757–200	2
Rolls-Royce Trent 556	Airbus	2003–2009	A340–600	2
Rolls-Royce Trent 772	Airbus	2009	A330–200	2
Rolls-Royce Trent 892	Boeing	2006	777–200	1
Rolls-Royce Trent 970	Airbus	2011–2013	A380–800	3
IAE V2522	Airbus	1998	A319–100	1
IAE V2524	Airbus	2005–2007	A319–100	2
IAE V2527	Airbus	2000–2013	A319–100, A320–200	17
IAE V2533	Airbus	2008–2014	A321–200	10
CFM, CFM International; GE, General Electric Aviation; PW, Pratt and Whitney; EA, Engine Alliance (GE/PW); IAE, International Aero Engines.				

**Table 4:** Summary statistics of selected engine emissions indices.

**Engine manufacturer, model, and series**	**Particle number (kg**^**−1**^)	**Non-volatile particle number (kg**^**−1**^)	**CCN number (kg**^**−1**^)	**BC-equivalent particle mass (mg kg**^**−1**^)	**Particle volume (mm**^**3**^ **kg**^**−1**^)
CFM CFM56-3B	3.56×10^16^⋇1.41	2.54×10^15^⋇2.35	7.28×10^14^⋇1.39	564⋇1.41	406⋇1.25
CFM CFM56-3C	1.65×10^16^	2.31×10^15^	6.85×10^14^	792	700
CFM CFM56-5A	4.62×10^16^⋇1.25	1.85×10^15^⋇2.50	5.41×10^14^⋇1.44	419⋇1.37	327⋇1.34
CFM CFM56-5B	5.09×10^16^⋇1.39	1.78×10^15^⋇2.20	4.51×10^14^⋇1.53	276⋇1.51	274⋇1.30
CFM CFM56-5C	5.06×10^16^⋇1.02	1.11×10^15^⋇1.55	4.54×10^14^⋇1.07	416⋇1.08	317⋇1.18
CFM CFM56-7B	5.22×10^16^⋇1.31	2.21×10^15^⋇2.33	4.55×10^14^⋇1.91	348⋇1.55	306⋇1.38
GE CF34-3	3.62×10^16^⋇1.50	4.99×10^15^⋇1.34	9.13×10^14^⋇1.23	488⋇1.59	361⋇1.39
GE CF34-8	2.98×10^16^⋇1.74	2.67×10^15^⋇1.79	6.97×10^14^⋇1.90	435⋇1.49	295⋇1.50
GE CF6-80C2	3.83×10^16^⋇1.45	1.06×10^15^⋇2.54	2.86×10^14^⋇1.66	190 ⋇ 1.49	203⋇1.57
GE GE90-94B	3.95×10^16^⋇1.16	5.12×10^14^⋇1.13	1.80×10^14^⋇1.27	92.8⋇1.12	124⋇1.40
GE GE90-115B	3.39×10^16^⋇1.20	7.95×10^14^⋇1.96	3.42×10^14^⋇1.58	175⋇1.30	191⋇1.33
GE GEnx-2B67	8.83×10^16^⋇1.41	1.95×10^15^⋇4.07	3.77×10^14^⋇1.50	46.1⋇1.48	557⋇1.34
EA GP7270	2.86×10^16^⋇1.25	1.89×10^15^⋇1.18	3.23×10^14^ ⋇ 1.16	129⋇1.33	171⋇1.04
PW JT8D	1.73×10^16^⋇2.89	2.83×10^15^⋇2.53	1.27×10^15^⋇1.55	941⋇1.06	617⋇1.23
PW PT6A	3.77×10^16^⋇1.84	6.50×10^15^⋇1.79	6.45×10^14^⋇2.07	441⋇2.05	458⋇2.02
PW PW118	5.80×10^16^⋇1.33	2.98×10^15^⋇1.69	8.80×10^14^⋇1.30	649⋇1.62	478⋇1.52
PW 150A	5.87×10^16^⋇1.26	4.49×10^15^⋇3.56	5.93×10^14^⋇1.02	252⋇1.51	356 ⋇ 1.32
PW PW2000	2.46×10^16^	2.55×10^15^	1.16×10^15^	817	650
PW PW4000	8.80×10^15^⋇1.06	1.70×10^15^⋇1.22	5.11×10^14^⋇1.48	439⋇1.65	341⋇1.55
Rolls-Royce AE3007	2.61×10^16^⋇3.11	1.45×10^15^⋇1.02	1.90×10^14^⋇3.27	592	138⋇1.32
Rolls-Royce RB211	4.12×10^16^⋇1.39	1.07×10^15^⋇2.08	2.25×10^14^⋇1.01	196⋇1.25	242⋇1.70
Rolls-Royce Trent 556	2.83×10^16^⋇1.28	1.06×10^15^ ⋇ 3.99	3.18×10^14^⋇1.31	319⋇1.49	212⋇1.28
Rolls-Royce Trent 772	2.49×10^16^⋇1.32	1.06×10^15^⋇1.37	3.09×10^14^⋇1.21	177⋇1.01	171⋇1.39
Rolls-Royce Trent 892	3.04×10^16^	8.09×10^14^	4.36×10^14^	304	249
Rolls-Royce Trent 970	5.13×10^16^⋇1.17	8.44×10^14^⋇1.60	3.12×10^14^⋇1.23	356⋇1.23	340⋇1.10
IAE V2522	2.58×10^16^	2.86×10^15^	1.02×10^15^	584	392
IAE V2524	1.14×10^16^⋇1.15	3.42×10^15^⋇2.10	1.44×10^15^⋇1.43	786⋇1.21	501⋇1.16
IAE V2527	1.93×10^16^⋇1.75	2.36×10^15^⋇1.73	8.29×10^14^⋇1.51	414⋇1.33	376⋇1.36
IAE V2533	2.09×10^16^⋇1.98	1.90×10^15^⋇3.15	5.68×10^14^⋇1.51	343⋇1.29	289⋇1.50
Particle number measured by a TSI 3775 condensation particle counter (particle diameters, D_p_>4 nm); non-volatile particle number measured by a TSI 3022 condensation particle counter (D_p_>7 nm); cloud condensation nuclei (CCN) number concentration at (2.6±0.2)% supersaturation measured by a DMT CCN counter; black-carbon-equivalent (BC-equiv.) particle mass measured by a Thermo Multi-Angle Absorption Photometer (MAAP); particle volume measured by a TSI Engine Exhaust Particle Sizer (EEPS) (5.6 nm>D_p_>560 nm). Emissions indices reported as the geometric mean ⋇ 1 geometric standard deviation (g.s.d). Note that kg^**−**1^ denotes ‘per kilogram of fuel’.					

**Table 5:** Log-normal fit coefficients for particle number and volume size distributions.

**Engine manufacturer, model, and series**	**Particle Number Emissions Index**						**Particle Volume Emissions Index**						**No. of Plumes**
	**N**_**1**_	**D**_**gN,1**_	**σ**_**N1**_	**N**_**2**_	**D**_**gN,2**_	**σ**_**N2**_	**V**_**1**_	**D**_**gV,1**_	**σ**_**V1**_	**V**_**2**_	**D**_**gV,2**_	**σ**_**V2**_	
CFM CFM56-3B	2.13×10^16^	12.9	1.32	3.90×10^15^	34.8	1.77	37	17.3	1.37	354	84.0	1.76	17
CFM CFM56-3C	6.39×10^16^	11.0	1.21	6.00×10^15^	36.4	2.02	20	21.4	2.36	692	98.4	1.61	1
CFM CFM56-5A	3.09×10^16^	13.9	1.38	1.14×10^15^	52.3	1.59	85	20.8	1.47	221	93.9	1.60	10
CFM CFM56-5B	3.59×10^16^	14.7	1.41	5.48×10^15^	61.7	1.45	118	22.6	1.48	131	95.0	1.48	37
CFM CFM56-5C	3.72×10^16^	14.5	1.39	6.81×10^15^	66.3	1.46	115	21.8	1.47	202	103	1.47	2
CFM CFM56-7B	3.61×10^16^	14.7	1.42	8.38×10^14^	50.9	1.59	124	23.1	1.49	147	96.5	1.54	80
GE CF34-3	2.03×10^16^	12.5	1.34	2.73×10^15^	41.3	1.61	49	20.8	1.54	289	77.0	1.64	6
GE CF34-8	2.08×10^16^	12.8	1.35	2.11×10^15^	38.9	1.66	47	19.7	1.50	212	78.0	1.70	21
GE CF6-80C2	3.37×10^16^	14.8	1.40	3.46×10^14^	54.9	1.68	112	22.4	1.47	73	103	1.46	5
GE GE90-94B	2.83×10^16^	13.7	1.36	2.37×10^14^	54.5	1.60	73	20.0	1.46	47	98.2	1.50	2
GE GE90-115B	2.79×10^16^	14.9	1.39	3.53×10^14^	59.9	1.59	85	21.4	1.43	95	109	1.53	5
GE GEnx-2B67	4.79×10^16^	22.1	1.52	—	—	—	518	34.7	1.46	—	—	—	2
EA GP7270	3.44×10^16^	14.4	1.39	2.44×10^14^	65.5	1.41	101	21.2	1.46	59	92.9	1.39	3
PW JT8D	6.07×10^15^	12.9	1.36	5.43×10^15^	38.8	1.91	31	27.4	1.42	590	91.5	1.50	2
PW PT6A	2.08×10^16^	14.4	1.51	3.90×10^15^	38.6	1.63	77	26.9	1.48	370	81.2	1.70	5
PW PW118	4.26×10^16^	13.9	1.40	1.06×10^15^	59.1	1.58	141	23.4	1.55	266	105	1.52	10
PW 150A	2.61×10^16^	12.2	1.27	5.96×10^15^	22.0	1.91	43	16.2	1.40	231	77.2	1.92	2
PW PW2000	2.52×10^16^	14.8	1.41	2.30×10^15^	58.6	1.58	91	23.6	1.50	578	104	1.53	1
PW PW4000	4.52×10^16^	11.6	1.29	3.18×10^15^	38.2	1.87	14	22.8	1.56	332	90.1	1.55	2
Rolls-Royce AE3007	3.39×10^16^	12.9	1.34	5.80×10^14^	24.7	2.47	85	20.1	1.56	66	139	1.47	2
Rolls-Royce RB211	2.69×10^16^	14.6	1.42	1.81×10^15^	26.4	2.08	106	24.4	1.56	124	110	1.54	2
Rolls-Royce Trent 556	1.86×10^16^	12.7	1.32	5.06×10^14^	62.0	1.58	41	18.7	1.53	168	121	1.58	2
Rolls-Royce Trent 772	2.38×10^16^	13.9	1.37	6.14×10^14^	45.8	1.74	69	21.0	1.51	94	102	1.52	2
Rolls-Royce Trent 892	3.79×10^16^	15.3	1.41	2.35×10^14^	78.8	1.35	128	22.5	1.43	125	120	1.54	1
Rolls-Royce Trent 970	3.86×10^16^	16.4	1.45	3.00×10^14^	83.1	1.46	160	24.5	1.42	181	131	1.50	3
IAE V2522	1.64×10^16^	11.6	1.25	3.74×10^15^	36.4	1.83	30	16.7	1.56	375	91.9	1.62	1
IAE V2524	3.62×10^15^	10.7	1.23	4.91×10^15^	40.2	1.63	—	—	—	477	81.6	1.58	2
IAE V2527	1.52×10^16^	12.3	1.29	4.62×10^15^	30.5	1.92	37	19.1	1.51	319	85.1	1.59	16
IAE V2533	1.94×10^16^	12.1	1.28	3.42×10^15^	26.2	1.99	36	16.8	1.44	211	91.6	1.72	9
All Sampled Plumes (as shown in Fig. 6)	**3.58×10**^**16**^	**12.7**	**1.56**	**6.40×10**^**14**^	**61.1**	**1.48**	**95.1**	**23.2**	**1.56**	**153**	**96.9**	**1.48**	**253**
